# *Anisandrusbolavenensis* sp. nov. (Col.: Curculionidae, Scolytinae, Xyleborini), a new ambrosia beetle from Laos

**DOI:** 10.3897/BDJ.12.e130023

**Published:** 2024-07-18

**Authors:** Wisut Sittichaya, Sarah M. Smith

**Affiliations:** 1 Agricultural Innovation and Management Division, Faculty of Natural Resources, Prince of Songkla University, 90110, Songkhla, Thailand Agricultural Innovation and Management Division, Faculty of Natural Resources, Prince of Songkla University, 90110 Songkhla Thailand; 2 Department of Entomology, Michigan State University, 288 Farm Lane, 243 Natural Science Bldg., East Lansing, MI 48824, United States of America Department of Entomology Michigan State University, 288 Farm Lane, 243 Natural Science Bldg., East Lansing, MI 48824 United States of America

## Abstract

**Background:**

The ambrosia beetle genus *Anisandrus* Ferrari, 1867, is a member of the bark and ambrosia beetle subfamily Scolytinae, Tribe Xyleborini. Currently, it is comprised of 40 species of which four species were recorded in Laos.

**New information:**

A new species, *Anisandrusbolavenensis* **sp. nov.** is described from the Bolaven Plateau in southern Laos. With the inclusion of the species described and recorded here, the diversity of *Anisandrus* is increased to 41 species, of which five occur in Laos. New distribution records, a synoptic list and a key to the *Anisandrus* of Laos PDR are presented.

## Introduction

The ambrosia beetle genus *Anisandrus* Ferrari, 1867, is one of the more diverse members of the tribe Xyleborini in Asia and is currently comprised of 40 species of which 33 are distributed in Indochinese Peninsula and China (Smith et al. 2020, Smith et al. 2022; Sittichaya et al. 2023). Sittichaya et al. (2023) provided distributions and a short taxonomic history of the genus. Generic diagnostic characters are given by Smith et al. (2020) and subsequently modified by Sittichaya et al. (2023). Four *Anisandrus* species were previously recorded from Laos (Beaver et al. 2014, Beaver and Liu 2018, Smith et al. 2020). In the present study, we describe one new species from Laos, increasing the diversity of the Laos fauna to five species and that of the genus to 41. We also present new records and provide a key and synoptic list of the *Anisandrus* of Laos.

## Materials and methods

Specimens were extracted from a small branch of *Symplocos* sp. (Symplocaceae) from the Bolaven Plateau, Champasak Province, Laos. The new species was then compared with types specimens or photos of *Anisandrus* type specimens (in MSUC, NMNH, NHMUK, NHMW, WSTC) photographed by the authors. Type material of the great majority, over 90%, of described *Anisandrus* have been examined by at least one of the authors. Photographs of type specimens of almost all the remaining described species were also examined by at least one author. Photographs were processed as described in Sttichaya et al. (2024). Antennal club and pronotum types and characters follow those proposed by Hulcr et al. (2007) and subsequently elaborated by Smith et al. (2020). The number of segments in the antennal funicle excludes the pedicel. Length was measured from the pronotum apex to the apex of the declivity excluding processes or spines and width at the widest part of specimen.

**Abbreviations used for entomological collections**

- MSUC, Albert J. Cook Arthropod Research Collection, Michigan State University, East Lansing, USA
- NHB, Naturhistorisches Museum Basel, Switzerland
- NHMUK, Natural History Museum, London, UK
- NMNH, Museum of Natural History, Washington, D.C., USA
- NHMW, Naturhistorisches Museum Wien, Austria
- WSTC, Private collection of Wisut Sittichaya, Songkhla, Thailand

## Taxon treatments

### Anisandrus bolavenensis sp. nov.

F4B22EC5-56CF-55C0-BB04-255AD98DF8FB

C7592BD6-6FA5-48FA-8E7F-386895E9C0F0

#### Materials

- **Type status:** Holotype. **Occurrence:** recordNumber: 1; recordedBy: Sittichaya & Smith; individualCount: 1; sex: female; lifeStage: Adult; disposition: NHMW; occurrenceID: 7AE477B9-F5C5-50BB-8418-2D42EE1EA5F7; **Location:** country: Laos PDR; stateProvince: Champasak; county: Paksxong; locality: Bolaven plateau; verbatimLocality: Paksong; verbatimElevation: 1150 m; verbatimCoordinates: 15°04'26.4"N 106°12'03.3"E; **Identification:** identifiedBy: Sittichaya, W & Smith SM; **Event:** samplingProtocol: hand collecting (W. Sittichaya); eventDate: 14.viii.2023; **Record Level:** type: Holotype

#### Description

**Figure 1.**
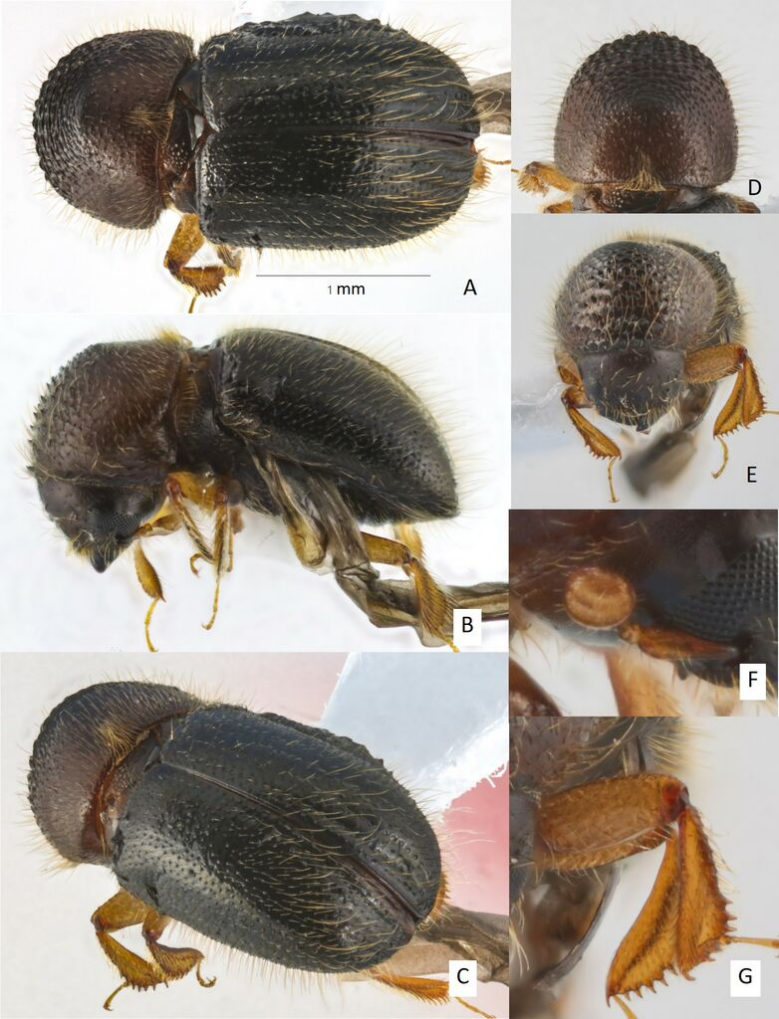
*Anisandrusbolavenensis* sp. nov. holotype female **A** dorsal view; **B** lateral view; **C** postero-lateral view; **D** pronotum; **E** frons; **F** antenna; **G** pro- and mesotibiae.

**Female**. (Fig. 1) 2.4 mm long; 1.71× as long as wide. Body less elongate, stout. Head, anterior slope of pronotum and ventrite dark brown, pronotal disc reddish-brown, elytra black and appendages brown. Body densely covered with different long brownish setae. **Head**: epistoma complete, transverse, with a row of short, hair-like setae, setae moderately dense. Frons feebly impressed from epistoma to upper margin of eyes, impressed areas sub-rectangular, deepest at epistoma, the depth decreasing upwardly to upper margin of the eyes, surface of impressed areas feebly convex, alutaceous, strongly shiny, rather sparsely punctured, puncture small and shallow, some larger punctures near lateral margin of the eyes, each puncture with very long brownish, fine, hair-like setae; a weak, impunctate median ridge extends to upper level of eyes. The area from mandible bases to lateral margin of eyes weakly convex, punctate; punctures broad and shallow. Eyes feebly emarginate just above antennal insertion, upper part slightly smaller than lower part. Submentum triangular, small, slightly impressed. Antennal scape long and thickened, 1.12× as long as club. Pedicel as wide as scape, shorter than funicle. Funicle 4-segmented, segment 1 as long as pedicel. Club longer than wide, obliquely truncate, type 1; segment 1 corneous, encircling anterior face, anterior margin concave, sharp carinate; segment 2 corneous on anterior face, corneous part concave, narrow; sutures absent on posterior face. **Pronotum**: 0.94× as long as wide. In dorsal view, type 0, feebly conical anteriorly, sides convex; anterior margin with a row of seven small, slightly protruding serrations, equal in size to those on anterior slope. In lateral view, type 3, short and tall; disc as long as anterior slope, summit at mid-point. Anterior slope with moderately densely spaced, large coarse asperities, becoming lower and more strongly transverse towards summit; setose, setae moderately long, semi-recumbent, pointing backwards, decreasing in length to summit. Disc alutaceous, shiny, with moderately dense, fine granulate punctures, each puncture either with a short, fine, semi-recumbent, brownish hair-like seta or with long semi-erect brownish hair-like seta, the longer setae 2–3× as long as the short setae, setae pointing anteriorly, some longer setae present at margins. Lateral margins obliquely costate. Base slightly concave, posterior angles angularly rounded. Mycangial tuft present along basal margin, tuft moderately setose, approximately the width of scutellum. **Elytra**: 1.28× as long as wide, 1.78× as long as pronotum. Scutellum broad, large, linguiform, flush with elytra, flat, shiny. Elytral base transverse, edge oblique, humeral angles rounded, parallel-sided in basal 2/3, then broadly rounded to apex. Disc strongly shiny, broadly convex, striae flat, with small, shallow, oblique margin, setose punctures separated by < 0.5× diameter of a puncture, setae 2–2.5× as long as diameter of punctures, fine, recumbent, hair-like; interstriae flat, 2× as wide as striae, granulated punctures confused, setose; setae long, 2× as long as strial setae, erect hair-like, increasing their length and thickness posteriorly; granules in interstrial punctures minute, but distinct. Declivity occupying approximately half of elytral length, densely setose, summit broadly rounded to declivity, declivital face flattened from interstriae 1–3, feebly convex near lateral margins from interstriae 4–6; striae distinctly impressed; strial punctures shallow, 2× larger than those of disc, striae 1–3 curved laterally, striae with fine recumbent, hair-like setae, setae 2–2.5× as long as diameter of punctures, pointing inwardly to declivital medial suture; interstriae uniseriate granulated; granule minute, setose, setae very long, 3–5× width of an interstria, erect, hair-like. Posterolateral margin rounded, unarmed by granules, costate only close to apex. **Legs**: procoxae contiguous. Protibiae obliquely triangular, broadest at apical 1/3; posterior face inflated, minutely granulate; apical 1/3 of outer margin with six small, socketed denticles, their length as long as basal width. Meso- and metatibiae flattened; outer margins evenly rounded with nine and eight long and slender, acute, socketed denticles, respectively.

**Male.** Unknown.

##### Similar species

*Anisandrusauco* Smith, Beaver & Cognato, 2020, *A.cryphaloides* Smith, Beaver & Cognato, 2020, *A.tanaosi* Sittichaya, Smith & Beaver, 2023.

#### Diagnosis

2.4 mm long (n = 1); 1.71× as long as wide. Small and stout species. Pronotal anterior margin slightly angularly projecting, median pair of asperities on anterior margin not prominent; elytral disc broadly convex, strongly shiny, without a saddle-like impression, declivity appears more obliquely sloped as compared to many other *Anisandrus* species, declivital summit unarmed, declivity flat from interstriae 1–3, declivity unarmed, densely covered with interstrial setae, setae very long, 3–4× as long as interstrial width; striae distinctly impressed; strial punctures shallow, 2× larger than those of disc, striae with fine recumbent, hair-like setae, setae 2–2.5× as long as diameter of punctures, setae pointing inward to declivital medial suture.

This species is similar to *A.auco*, *A.cryphaloides* and *A.tanaosi*. It can be distinguished from *A.auco* by the following characters (*A.bolavenensis* given first): declivital face flattened from interstriae 1–3, feebly convex near lateral margins from interstriae 4–6 and posterolateral margin near the apex costate vs. declivital face convex and posterolateral margin rounded along its length.

It is distinguished from *A.cryphaloides* by the following characters (*A.bolavenensis* given first): pronotum from dorsal view feebly conical anteriorly, declivital summit unarmed and declivital face flattened vs. pronotum from dorsal view strongly protruding, declivital summit armed with a pair of small incurved spines on interstriae 2 and declivital face convex.

It is also distinguished from *A.tanaosi* by the following characters (*A.bolavenensis* given first): declivital summit unarmed vs. declivital summit armed with a pair of spinulose granules on interstriae 2.

#### Etymology

Bolavenensis, in reference to the collection locality of the holotype, Bolaven Plateau, Lao People's Democratic Republic. Noun in apposition.

#### Distribution

Laos (Champasak Province).

#### Biology

The holotype was collected from a newly fallen (leaves still green) small branch of *Symplocos* sp. (Symplocaceae).

### Anisandrus hirtus

(Hagedorn, 1904)

43E2B34E-A727-5A10-84D2-AB5C5845DDFE

#### Materials

- **Type status:** Other material. **Occurrence:** recordedBy: Vít Kubáň; individualCount: 12; sex: female; lifeStage: Adult; disposition: NHB; occurrenceID: 7D5E05D6-E0BE-5B50-9D09-13014788C385; **Location:** country: Laos PDR; stateProvince: Hua Phan; locality: Phu Phan Mt.[mountain]; verbatimElevation: ~ 1750 m; verbatimCoordinates: 20°12'N, 104°01'E; **Identification:** identifiedBy: Smith S.; **Event:** eventDate: 03.vi.2007

#### Description

3.4–4.5 mm long (mean = 3.92 mm; n = 5); 1.95–2.53× as long as wide. Body medium size, stout, brown to black in colour, appendages paler; densely covered with fine, long, erect dark brown hair-like setae. Pronotum from dorsal view round (type 1), base with mesonotal mycangial tuft the length of the scutellum; elytral disc convex; lateral side parallel to apical ¼ then rounded to apex; declivity rounded, summit unarmed, posterolateral margins rounded.

#### Distribution

Bhutan, Cambodia, China (Fujian, Guangxi, Jiangxi, Sichuan, Xizang, Yunnan), India (Meghalaya, West Bengal), Laos, Myanmar, Nepal, Taiwan,Thailand, Vietnam, New to Laos (Hua Phan).

### Anisandrus ursulus

(Eggers, 1923)

05992FAC-6115-5091-96CA-15845257070B

#### Materials

- **Type status:** Other material. **Occurrence:** recordNumber: 2; recordedBy: Vít Kubáň; individualCount: 2; sex: female; lifeStage: adult; disposition: NHB; occurrenceID: B559B261-0364-5775-9792-ED7165E15366; **Location:** country: Laos PDR; stateProvince: Hua Phan; locality: Phu Phan Mt.[mountain]; verbatimElevation: ~ 1750 m; verbatimCoordinates: 20°12'N, 104°01'E; **Identification:** identifiedBy: Smith S.; **Event:** eventDate: 03.vi.2007
- **Type status:** Other material. **Occurrence:** recordedBy: Vít Kubáň; individualCount: 1; occurrenceID: 9141C501-CFAB-50FA-AA98-01B85AEADFAD; **Location:** country: Laos; stateProvince: Phongsaly; locality: Phongsaly env.; verbatimElevation: ~ 1500 m; verbatimCoordinates: 21°41-2'N, 102°06-8'E; **Identification:** identifiedBy: Smith S; **Event:** eventDate: 8.v.-20.vi.2003
- **Type status:** Other material. **Occurrence:** recordedBy: M. Brancucci; individualCount: 1; sex: female; lifeStage: adult; disposition: NHB; occurrenceID: FCE80182-237B-57A6-A6A3-1FEE7110A664; **Location:** country: Laos; stateProvince: Phongsaly; locality: Phongsaly env.; verbatimElevation: ~ 1500 m; verbatimCoordinates: 21°41'N, 102°6'E; **Identification:** identifiedBy: Smith s; **Event:** eventDate: 6.-17.v.2004
- **Type status:** Other material. **Occurrence:** recordedBy: D. Hauck; individualCount: 2; sex: female; lifeStage: adult; disposition: NHB; occurrenceID: 9933E070-0D18-5619-A114-C6FEFEA8BCFF; **Location:** country: Laos; stateProvince: , Phongsaly env., 6.-17.v.2004, ~1500m, M. Brancucci leg. (1, NHB). Xieng; locality: Phonsavan (30 km NE): Phou Sane Mt.; verbatimElevation: 1420 m; verbatimCoordinates: 19°38.20'N, 103°20.20'E; **Identification:** identifiedBy: Smith S; **Event:** eventDate: 10.-30.v.2009

#### Description

4.3–4.9 mm long (mean = 4.5 mm; n = 5); 1.88–1.96× as long as wide. Body large size, robust and stout, brown to black in colour, appendages paler; densely covered with fine, moderately long, erect, dark brown hair-like setae. Pronotum from dorsal view round (type 1) anterior margin armed with 5 serrations, the middle pair is prominent; pronotal base with mesonotal mycangial tuft the length of the scutellum; elytra short, disc convex; lateral side parallel to apical 1/3 then broadly rounded to apex; declivity obliquely truncate, unarmed, declivital striae not impressed and posterolateral margins obliquely truncate armed with small tubercles.

#### Distribution

China (Fujian, Guangdong, Guangxi, Jiangxi), India (Nicobar Is, West Bengal), Indonesia (Bali, Batoe Is, Java, Maluku, Sulawesi, Sumatra), Laos, East and West Malaysia, New Guinea, Philippines, Solomon Islands, Thailand, Vietnam (Smith et al. 2020). New to Laos (Hua Phan, Phongsaly, Xieng Khouang).

## Identification Keys

### Key to *Anisandrus* species present in Laos (females only)

**Table:** 

1	Interstriae 2 without spines or granules on upper margin of elytral declivity.	2
–	Interstriae 2 with spines, spinulose granules or blunt tubercles on upper margin of elytral declivity; sparsely hairy species.	4
2	Median pair of asperities on anterior margin of pronotum distinctly larger than outer pair, larger, 3.4‒4.9 mm long.	3
–	Median pair of asperities on anterior margin of pronotum not distinctly larger than outer pair(s), smaller, 2.4 mm long.	*A.bolavenensis* sp. nov.
3	Larger, stouter species, 4.3‒4.9 mm long, 1.9‒2.0× longer than wide; declivital striae not impressed.	*A.ursulus* (Eggers)
–	Smaller, more elongate species, 3.4‒4.5 mm long, 2.1‒2.5× longer than wide; declivital striae impressed.	*A.hirtus* (Hagedorn)
4	Posterolateral margins of elytra rounded; larger, 5.8–5.9 mm.	*A.niger* (Sampson)
–	Posterolateral margins of elytra costate or carinate; smaller, 3.3‒3.7 mm.	*A.cristatus* (Hagedorn)

## Discussion

The ambrosia beetle genus *Anisandrus* diversified in mountainous areas of Southeast Asia, particularly in the Indochinese Peninsula (Smith et al. 2020, Smith et al. 2022, Sittichaya et al. 2023). Laos is rich on natural habitats, 40% of the land being covered with forests and most of them are at high elevations (Chouangthavy et al. 2020). Four of five Laotian species (Table 1) were reported only in the north of the country and one from the south. There is no doubt that much of the diversity awaits discovery as its neighbours Thailand and Vietnam each have 11 species ([Bibr B11731499][Bibr B11731508][Bibr B11731490]).

## Supplementary Material

XML Treatment for Anisandrus bolavenensis

XML Treatment for Anisandrus hirtus

XML Treatment for Anisandrus ursulus

## Figures and Tables

**Table 1.** Synoptic list and habitat types of the *Anisandrus* fauna of Laos PDR. References are to records of the species in Laos PDR.

**Species**	**Laos provincial distribution**	**Habitat**	**Reference**
*Anisandrusbolavenensis* sp. nov.	Champasak	Bolaven Plateau, low montane forest, 1150 m	This publication
*Anisandruscristatus* (Hagedorn, 1908)	Hua Phan	Phou Pan (Mt.), 1300 –1900 m	Smith et al. (2020)
*Anisandrushirtus* (Hagedorn, 1904)	Phongsaly	Phu Phan Mt, ~ 1750 m	Beaver and Liu (2018), (personal communication), this publication
*Anisandrusniger* (Sampson, 1912)	Hua Phan	Phou Pan (Mt.), 1300–1900 m	Beaver and Liu (2018), Smith et al. (2020)
*Anisandrusursulus* (Eggers, 1923)	Dudomxai, Hua Phan Luang Phrabang, Xieng Khouang	Phu Phan Mt., ~ 1750 m, Phou Sane Mt., 1420 m	Beaver et al. (2014), this publication
